# Large Differences in Publicly Visible Health Behaviours across Two Neighbourhoods of the Same City

**DOI:** 10.1371/journal.pone.0021051

**Published:** 2011-06-09

**Authors:** Daniel Nettle

**Affiliations:** Centre for Behaviour and Evolution, Institute of Neuroscience, Newcastle University, Newcastle, United Kingdom; Pennington Biomedical Research Center, United States of America

## Abstract

**Background:**

There are socioeconomic disparities in the likelihood of adopting unhealthy behaviours, and success at giving them up. This may be in part because people living in deprived areas are exposed to greater rates of unhealthy behaviour amongst those living around them. Conventional self-report surveys do not capture these differences in exposure, and more ethological methods are required in order to do so.

**Methodology/Principal Findings:**

We performed 12 hours of direct behavioural observation in the streets of two neighbourhoods of the same city which were similar in most regards, except that one was much more socioeconomically deprived than the other. There were large differences in the publicly visible health behaviours observed. In the deprived neighbourhood, we observed 266 more adults smoking (rate ratio 3.44), 53 more adults drinking alcohol (rate ratio not calculable), and 38 fewer adults running (rate ratio 0.23), than in the affluent neighbourhood. We used data from the Health Survey for England to calculate the differences we ought to expect to have seen given the individual-level socioeconomic characteristics of the residents. The observed disparities between the two neighbourhoods were considerably greater than this null model predicted. There were also different patterns of smoking in proximity to children in the two neighbourhoods.

**Conclusions/Significance:**

The differences in observed smoking, drinking alcohol, and physical activity between these two neighbourhoods of the same city are strikingly large, and for smoking and running, their magnitude suggests substantial area effects above and beyond the compositional differences between the neighbourhoods. Because of these differences, individuals residing in deprived areas are exposed to substantially more smoking and public drinking, and less physical activity, as they go about their daily lives, than their affluent peers. This may have important implications for the initiation and maintenance of health behaviours, and the persistence of health inequalities.

## Introduction

In developed countries, people of lower socioeconomic position (SEP) are likely to engage in behaviours inimical to health, such as cigarette smoking [1], [2], [3], [4] and heavy episodic drinking of alcohol [5], [6], and less likely to engage in healthy behaviours such as physical exercise [7]. SEP also predicts the trajectory of change in health behaviour. Amongst those who attempt to give up smoking, people of lower SEP are much more likely to fail [8], and amongst those who exercise, people of lower SEP are more likely to desist over time [9]. There is also some evidence, at least among women, that childhood SEP can predict smoking and failure to quit even when adult SEP is controlled for [4].

Part of the reason that people of lower SEP are more likely to find it difficult to maintain healthy behaviour may be that they are more regularly exposed to other people around them modelling the unhealthy alternatives. For the case of smoking, for example, we know that having other smokers in one's social network promotes smoking maintenance and makes attempts to quit more likely to fail [10], [11], [12]. More generally, social psychologists have shown that behaviour (including health behaviour [13]) can be powerfully influenced by perceptions of what is locally normative [14], [15], [16]. These perceptions will be affected by the rates of behaviours observable in the surrounding population. If observable cues of drinking and smoking are particularly abundant in deprived areas, whilst cues of physical activity are particularly scarce, this would be one mechanism by which area effects – the predictive effects of neighbourhood deprivation on health behaviours above and beyond the individual-level characteristics of the people living there [17], [18] – could be generated.

Cross-sectional surveys (e.g. [3]) demonstrate social gradients in health behaviours, but they tend to be based on self-report, which may limit their reliability. Moreover, they do not allow us to fully quantify social differences in exposure to unhealthy behaviour, even when they include place of residence information. This is because such parameters as the extent to which the relevant behaviours occur out of doors, and the extent to which they happen in company rather than alone, may differ between poor and affluent areas. Thus, to understand what people living in different areas are exposed to, it may be useful to complement existing quantitative [3] and qualitative [19], [20] methodologies with more ethological approaches based on directly observing behaviour in context. Here, we report the results of such an observational study, in which we quantified the occurrence of public smoking, drinking of alcohol, and (as an example of physical activity) running in two contrasting neighbourhoods of the city of Newcastle upon Tyne.

As the UK has excellent national self-report surveys on health behaviour and a national census with a fine degree of spatial resolution, we were able to first calculate a ‘null model’ of what differences in behaviour we should expect given the individual-level socioeconomic characteristics of the residents of the two areas. The null model gives us a prediction of what we would observe if there are no area effects, that the self-report survey data faithfully represent actual behaviour, and that behaviours are equally likely to be performed in public in the two areas. Any discrepancies between the null model and what we actually observe will suggest that one or more of these assumptions is incorrect.

## Methods

### Ethics statement

All individuals observed were in public spaces where they would have expected their behaviour to be visible to others. No personally identifying information was recorded, and the researcher, though never questioned, was ready to explain the nature of the study and his purpose to any individual concerned. The study was approved by the Faculty of Medical Sciences ethics committee, Newcastle University.

### Study sites

We selected two areas within the city of Newcastle upon Tyne which were similar in size, centrality, ethnic composition and layout, each consisting of a main shopping street backed on either side by streets of terraced houses and flats. Each area was made up of two contiguous census Lower Super Output Areas (Area A = Newcastle upon Tyne 005C plus 006E; Area B = 27D plus 29B).The distance between the centres of the two areas was approximately 5.5 km. Their populations are similar in many ways, but socioeconomic variables are highly contrasting (Table 1), with Area B in the most socioeconomically deprived 1% of all English census areas, and Area A at the 79% percentile by the same measure.

**Table 1:** Comparison of the two study areas.

	*Area A*	*Area B*
Total population (males)	3098 (1502)	3223 (1508)
Children	710	808
Median age	37	34.5
Households	1250	1589
Population born in UK (%)	92	92
Index of Multiple Deprivation	8.74	76.43
Index of Multiple Deprivation, percentile of English neighbourhoods	79^th^	1^st^
Households owner-occupied (%)	83	18
Residents in highest socioeconomic group	74%	16%

Note: All figures are averages for the two constituent census LSOAs of each area. Sources: 2001 census and 2004 indices of multiple deprivation. IMD percentile is of all English census LSOAs, where 1^st^ represents the most deprived 1%.

### Sampling

We recorded data for every minute of a composite day (9am to 9pm) in each area, by dividing the time into 30 minute segments, and recording one segment on each available weekday from each area over 19^th^ April–8^th^ July 2010. Segments were completed in random order, but once a particular time of day had been sampled in one area, it was sampled in the other area as soon as possible (median delay 1 day; maximum 4 days). All five weekdays were represented at least 3 times in the data for each area. The researcher spent the first ten minutes of each segment walking the complete length of the main shopping street, and the remaining 20 minutes walking at normal speed along randomly varying routes through the residential streets.

### Data recording

The researcher wore a digital voice recorder and noted for each person encountered (i.e. passed within plain sight for long enough to be identified), whether they were a man, woman, child (estimated to be of statutory school age i.e. 16 or under), or baby (child not walking independently), the composition of social group in which they were moving, whether they were at that instant smoking, drinking an alcoholic drink from an open container, or running (no attempt was made to differentiate different reasons for running, and running was scored by gait alone rather than clothing). People inside buildings were not recorded, though those in open gardens or yards which were clearly visible from the street were. Individuals re-encountered within the same time segment were not re-recorded.

### Null model

To estimate the differences in health behaviours which we might expect to observe, we used the Health Survey for England 2008 (a nationally-representative survey of over 15,000 individuals [21]) to calculate the rate differences in self-reported smoking (proportion of individuals who smoke multiplied by number of cigarettes per day smoked by smokers), drinking alcohol (number of days in past week on which respondent had drunk alcohol), and running (proportion of people who had run in last two weeks multiplied by proportion of days on which runners had run) for adults across the three each occupationally-defined socio-economic groups (SEG-3). We then used the 2001 UK Census frequencies of adults from each socio-economic group residing in each of our study areas to calculate predicted rate ratios for observing adults smoking, drinking alcohol, and running, across the two areas.

## Results

In area A, we recorded 5884 people (4888 adults) in 4123 social groups. In area B, we recorded 6757 people (4750 adults) in 3773 social groups. The pattern of activity through the day was similar in the two areas, with the exception of larger numbers of people, especially children, observed in the residential streets of area B after 6pm.

### Smoking

The null model predicts that the rate of adult smoking will be 1.60 times higher in area B than area A. In fact, we observed 378 adults smoking (8.0% of adults observed) in area B as against 112 (2.3%) in area A (χ^2^ = 158.84, p<0.01), giving an observed rate ratio of 3.44. The discrepancy was more marked than in the residential than main streets (rate ratio 8.31 vs. 2.68). In addition, we observed 13 children smoking in area B, a behaviour not seen at all in area A.

**Figure 1:**
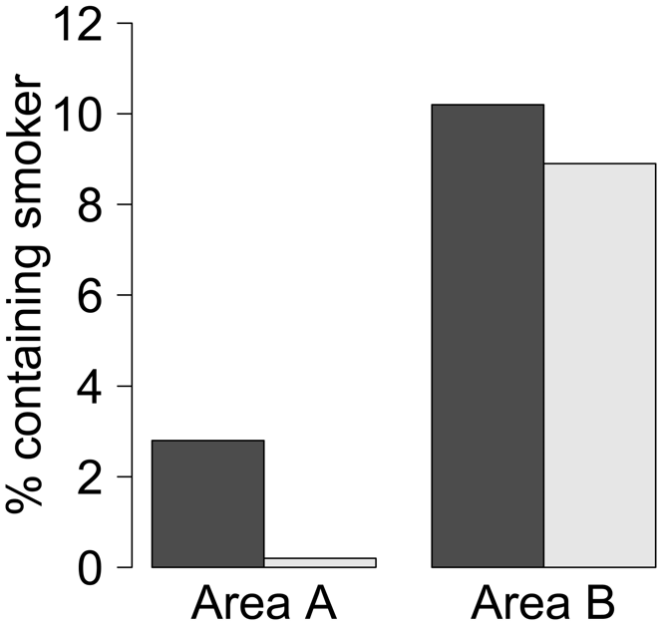
The percentage of social groups consisting of adults only (dark bars) or containing a minor (light bars) which contained a smoker, in areas A and B.

Considering social groups containing at least one adult, 2.5% contained a smoker in area A against 10.0% in area B. However, in area A, the probability of containing a smoker varied according to whether there was also a child or baby in the group: 2.8% (99/3540) in groups containing no minors, but 0.2% (1/435) where there were minors present (χ^2^ = 10.41, p<0.01). In area B, however, the likelihood of the social group containing a smoker was similar whether there were minors in the group or not (277/2710 or 10.2% no minors, 61/684 or 8.9% with minors, χ^2^ = 1.04, p = 0.31; Figure 1).

### Drinking alcohol

For drinking alcohol, the null model predicts that we should observe 0.77 times as much in area B as in area A (that is, mean alcohol consumption overall is actually higher amongst the higher socioeconomic groups, although heavy episodic drinking is not). In fact, no adults were observed drinking alcohol in area A, against 53 in area B (these were in 37 social groups in 14 of the 24 time segments). One child was observed drinking alcohol in area A, and 2 in area B. Adult drinking in area B was more frequent in residential than main streets (36 versus 17 adults; χ^2^ = 16.72, p<0.01), and concentrated in the evenings. Amongst social groups containing adults in area B, there was no less likely to be a drinker in the group if there were children present than if there were not (31 of 3003 (1.0%) groups with no children, 6 of 391 (1.5%) groups containing children; χ^2^ = 0.81, p = 0.37).

### Running

For running, the null model predicts a prevalence of adult running in area B 0.57 times that of area A. In fact, we observed 49 adult runners in area A against 11 in area B (rate ratio 0.23, χ^2^ = 23.14, p<0.01). We observed 3 children running in area A and none in area B. Runners were more common in residential than main streets in both areas, though the difference between residential and main streets was significant only in area A (data not shown).

**Figure 2:**
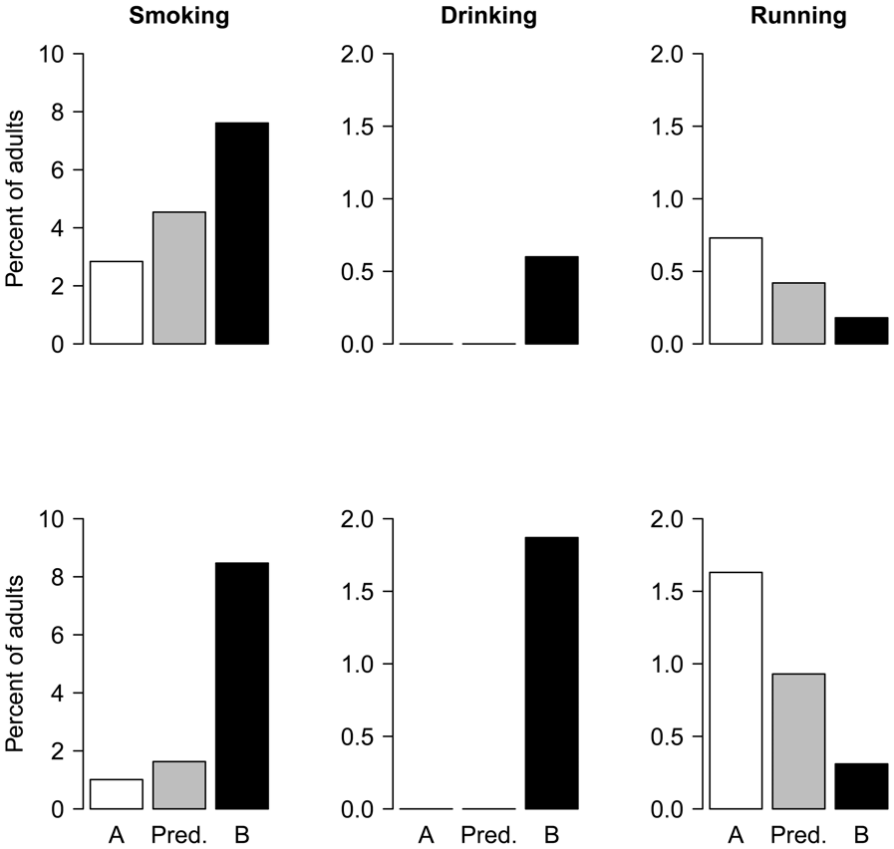
The percentage of adults smoking, drinking alcohol and running observed in area A (white bars), predicted for area B on the basis of the null model and the observed rate in area A (grey bars), and actually observed in area B (black bars). Note: The top row represents the main streets, and the bottom row the residential streets. The disparities between the two areas are in all cases much greater than predicted by the null model.

Figure 2 summarises the magnitude of the observed differences between the areas by showing, for each behaviour, the prevalence observed in area A (white bars), the prevalence predicted for area B given the null model and the observations in area A (grey bars), and the prevalence actually observed in area B. In every case, the observed difference is more than twice as great as the null model suggested. Table 2 summarises the differences observed across the two areas and types of streets, as rates of encounter per hour, rather than proportions of people observed.

**Table 2:** Average encounter rates per hour of people smoking, drinking alcohol, and running, for the two study areas.

	Main streets		Residential streets	
	Area A	Area B	Area A	Area B
Smoking	24.25	54.25	1.88	21.75
Drinking alcohol	0	4.25	0.13	4.75
Running	6.50	1.20	3.25	0.75

## Discussion

We observed very striking differences in publicly visible health behaviour in two neighbourhoods of the same city which were only 5.5 kilometres apart. In one day's worth of observation, we saw 266 more adults smoking, 53 more drinking, and 38 fewer running, in the deprived neighbourhood compared to the affluent one. These differences were not explained by the different numbers of people using the streets. Socioeconomic gradients in health behaviour are well known, and so there should be no surprise at the direction of the observed differences. However, their magnitude is in all cases much higher than the null model, which used the Health Survey for England, led us to predict (see Figure 2 for summary). The gulf between the null model and our observations was particularly marked in the residential streets. These may give the most accurate picture of the behaviour of the denizens of the two areas, since the main street of area A attracts many visitors from outside the neighbourhood, who come for the shops and restaurants there.

We do not claim to have comprehensively characterised the smoking, drinking and physical activity of the residents of the two neighbourhoods, since our methodology does not measure behaviour occurring indoors, or away from the neighbourhood. However, our results do provide a number of insights. For smoking and running, they suggest that there may be substantial area effects, given that the observed disparities were so much greater than those predicted by the null model. This conclusion is tentative for smoking, given that there could possibly be a greater rate of unobserved private smoking going on in the affluent neighbourhood, but firmer for running, where it is implausible to suggest that there is a great deal of running going on out of sight in the deprived neighbourhood. In addition, our data show that there are different norms regarding smoking in the company of children across the two neighbourhoods (see Figure 1), and this is suggestive of wider differences between social groups in attitudes towards smoking. For drinking alcohol, the data do not allow us to infer that overall consumption of alcohol is higher in area B than area A. In fact, the reverse is likely to be true, given that the higher socioeconomic groups have higher overall alcohol consumption. They do however reveal that the social setting of drinking behaviour is very different in the different neighbourhoods. This could be related to existing findings that bouts of heavy drinking, which is what has the greatest health impact, show the reverse gradient [6]. Thus, the ethological methods used here provide a rich source of insight into different local patterns of behaviour, and thus corroborate and extend existing qualitative approaches [19], [20].

Perhaps most importantly, we have provided a direct quantification of an important part of the experienced patterns of health behaviour which a person living in or other of these neighbourhoods is exposed to each day. Assume that a person spends one hour per day on the main street of the neighbourhood they live in, and one hour per day on the residential streets. Taking the encounter rates from Table 2, we can estimate that such a person would see around 18,000 more people per year smoking and 3,000 more people per year drinking on the streets if they lived in area B rather than A. A person living in area A, by contrast, would see around 2800 more people per year going for a run. These differences in exposure are likely to have substantial implications for initiation and maintenance of health behaviours, whether through changing perceptions of what is normative, or simply providing the visual suggestion of the behaviour.

In modern cities, individuals are spatially assorted by SEP to a remarkable extent, especially in countries with relatively high levels of inequality such as the UK and USA. If behaviours are influenced what people see going on around them as they go about their daily lives, and if the patterns observed here are generalizable beyond the two neighbourhoods of our study, social inequalities in health behaviour may prove stubbornly resistant to change. Clearly, social modelling of this kind cannot provide a complete explanation for social gradients in health behaviour (such an explanation would be circular, since it would basically argue that more people of low SEP smoke because more people of low SEP smoke). Some initial difference between social groups in outlook or decision-making is required for the social gradient to become established (see [22], [23], [24], [25] for discussion). However, once a social gradient exists, social modelling in a socioeconomically assorted urban environment can provide a powerful source of inertia entrenching prevailing patterns, and making it more difficult for individuals to change their behaviour. Effective health interventions will need to devise ingenious ways of overcoming this inertia.
